# Effect of particle size on the thermal conductivity of nanofluids containing metallic nanoparticles

**DOI:** 10.1186/1556-276X-6-247

**Published:** 2011-03-22

**Authors:** Pramod Warrier, Amyn Teja

**Affiliations:** 1 School of Chemical & Biomolecular Engineering, Georgia Institute of Technology, Atlanta, GA 30332-0100, USA

## Abstract

A one-parameter model is presented for the thermal conductivity of nanofluids containing dispersed metallic nanoparticles. The model takes into account the decrease in thermal conductivity of metal nanoparticles with decreasing size. Although literature data could be correlated well using the model, the effect of the size of the particles on the effective thermal conductivity of the nanofluid could not be elucidated from these data. Therefore, new thermal conductivity measurements are reported for six nanofluids containing silver nanoparticles of different sizes and volume fractions. The results provide strong evidence that the decrease in the thermal conductivity of the solid with particle size must be considered when developing models for the thermal conductivity of nanofluids.

## Introduction

Recent interest in nanofluids stems from the work of Choi et al. [1] and Eastman et al. [2], who reported large enhancements in the thermal conductivity of common heat transfer fluids when small amounts of metallic and other nanoparticles were dispersed in these fluids. Others [3-9] have also reported large thermal conductivity enhancements in nanofluids containing metal nanoparticles, although the effect of particle size, in particular, was not studied explicitly in these experiments. In our previous work [10-15], we have reported data for the thermal conductivity of nanofluids containing metal oxide nanoparticles, and critically reviewed [15] these and other data to determine the effect of temperature, base fluid properties, and particle size on the thermal conductivity of the nanofluids. These studies have led us to the conclusion that the temperature dependence of the nanofluid thermal conductivity arises predominantly from the temperature-thermal conductivity behavior of the base fluid, and that the effective thermal conductivity of nanofluids decreases with decreasing size of dispersed particles below a critical particle size. We have also presented a model [15] based on the geometric mean of the thermal conductivity of the two phases to predict the thermal conductivity of the heterogeneous nanofluid. The model incorporated the size dependence of the thermal conductivity of semiconductor and insulator particles using the phenomenological relationship proposed by Liang and Li [16]. The resulting 'modified geometric mean model' was able to predict the thermal conductivity of nanofluids containing semiconductor and insulator particles dispersed in a variety of base fluids over an extended temperature range. In the present work, we propose a similar geometric mean model that incorporates the size dependence of the thermal conductivity of metallic particles.

Previous experimental studies of nanofluids containing metallic particles employed very low volume fractions (<1%) of these particles. As a result, any size dependence of the thermal conductivity of the nanofluid was not apparent from these measurements and the data could be correlated using the bulk thermal conductivities of the solid and base fluid. We have now measured the thermal conductivity of nanofluids containing several volume fractions of silver nanoparticles of three sizes, and fitted the data with a model that incorporates the size dependence of the thermal conductivity of the solid phase. We show that such a model provides a better representation of the data than models that assume a constant (bulk) thermal conductivity for metallic particles of different sizes.

## The thermal conductivity of metallic nanoparticles

The kinetic theory expression [17] for the thermal conductivity *k*_b _of bulk metals is given by(1)

where *ρ *is the mass of electrons per unit volume, *C*_v,e _the volumetric specific heat of electrons, *v*_F _the Fermi velocity, and *λ*_e,b _is the mean free path of electrons in the bulk material. Substituting for electronic specific heat and Fermi velocity in Equation 1 leads to the relationship:(2)

where *n*_e _and *m*_e _are the number of free electrons per atom and the mass of an electron, respectively. These values are presented in Table 1 for a number of metals [17]. Equation 2 can be used to calculate the mean free path of electrons in the solid *λ*_e,b _if the bulk thermal conductivity and Fermi energy are known.

**Table 1:** Properties of metals at 298.15 K [17]

	***k***_**b**_**/W m**^**-1 **^**K**^**-1**^	***μ***_**F**_**/eV**	***n***_**e **_**10**^**28 **^**/m**^**-3**^	***λ***_**e,b**_**/nm**
Silver	424	5.51	5.85	49.10
Copper	398	7	8.45	35.97
Gold	315	5.5	5.9	36.14

Boundary or interface scattering will lead to a decrease in the electron mean free path and will become significant when the characteristic size *L *(= diameter of the particles) is of the same order as the electron mean free path. In this case, Equation 2 implies that the thermal conductivity of the particle will decrease with decreasing size. When *L *<<*λ*_e,b_, the thermal conductivity of the particle *k*_P _can be expressed as [17]:(3)

where *Kn *= *λ*_e,b_/*L *is the Knudsen number. When *L *is of the same order as *λ*_e,b_, the effective mean free path of the electron in the particle can be calculated using Matthiessen's rule:(4)

This leads to the following relationship for the thermal conductivity of the particle [17]:(5)

**Figure 1:**
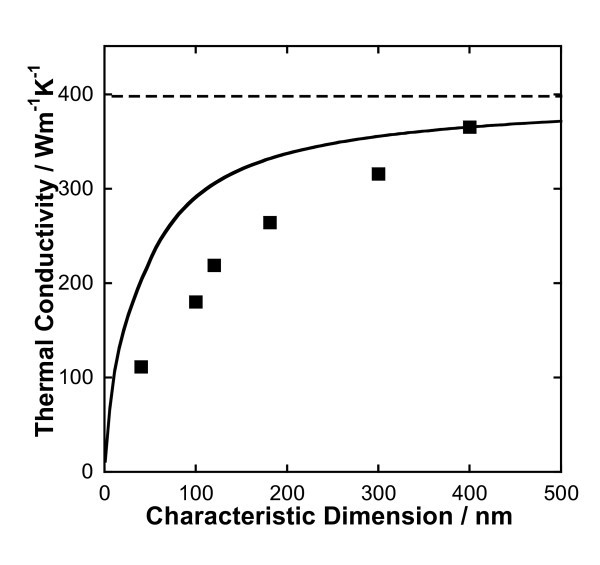
**Size dependent thermal conductivity of copper**. The *solid line *represents the thermal conductivity of copper nanoparticles calculated using Equations 3 and 5. The *dashed line *represents the bulk thermal conductivity of copper at 298 K. Data points are for copper thin films [18]

Equations 3 and 5 relate the thermal conductivity of metallic nanoparticles to their characteristic size, and is illustrated in Figure 1 for copper nanoparticles. The solid line in Figure 1 was obtained using Equation 3 to calculate the thermal conductivity when *Kn *> 5, and Eq. 5 when *Kn *< 1. In the intermediate region (1 <*Kn *< 5), the thermal conductivity was obtained by interpolation. Although no data are available to validate these calculations, the measurements of Nath and Chopra [18] for the thermal conductivity of thin films of copper (also plotted in Figure 1) clearly show a decrease in the thermal conductivity as the thickness of the film decreases. We expect metallic nanoparticles to exhibit similar trends with size. The dashed line in Figure 1 shows the bulk value of the thermal conductivity of copper, which is significantly higher than the measured values for thin films.

## Geometric mean model for the thermal conductivity of nanofluids

In our earlier work [13], we have shown that the thermal conductivity of nanofluids can be modeled using the Landau and Lifshitz [19] relation for the thermal conductivity of heterogeneous materials [20,21]:(6)

where *k*_eff_, *k*_p_, and *k*_l _are the thermal conductivities of the nanofluid, particles, and liquid, respectively, and φ is the volume fraction of the particles. For *n *= 1, this equation reduces to the arithmetic mean of the thermal conductivities of the two phases, which provides a good representation for conduction in materials arranged in parallel. Similarly, when *n *= -1, Equation 6 reduces to the harmonic mean of the two thermal conductivities, which provides a good representation for conduction in materials arranged in series. Finally, for *n *approaching zero, Equation 6 reduces to the geometric mean of the thermal conductivity of the two materials as follows:(7)

Turian et al. [20] have shown that Equation 7 works well for heterogeneous suspensions in which *k*_P_*/k*_l _> 4, whereas the Maxwell model [22] provides a lower bound for the thermal conductivity for dilute suspensions or when *k*_P_*/k*_l _~ 1. We have shown [15] that Equation 7 works well for thermal conductivity enhancement in nanofluids containing semiconductor and insulator particles if we account for the temperature dependence of *k*_l_, as well as the particle size and temperature dependence of *k*_P_. The modified geometric mean model may be expressed as:(8)

where *k*_eff_(*L*,*T*, φ) is the effective thermal conductivity of the nanofluid as a function of particle size (*L*), temperature (*T*), and particle volume fraction (φ), *k*_l_(*T*) is the thermal conductivity of the base fluid as a function of temperature, and *k*_P_(*L,T*) is the thermal conductivity of the particle as a function of particle size and temperature. In this work, we calculate *k*_P_(*L,T*) using Equations 3 and 5 as discussed in "The thermal conductivity of metallic nanoparticles" section. Equation 6 is used to fit measurements of the thermal conductivity of nanofluids with *n *as the adjustable parameter.

## Thermal conductivity of nanofluids

Literature data for nanofluids containing metallic nanoparticles were compiled and fitted using Equation 6 with and without considering the size dependence of the thermal conductivity of the particles. Table 2 lists our results for the two cases. Equation 6 is able to fit the literature data for nanofluids containing metallic particles reasonably well. However, values of *n *required to fit the data are higher than expected, and increase when the size dependence is considered. High values of *n *appear to be related to unusually large thermal conductivity enhancements. For example, enhancements of 80% were reported for 0.3% (v/v) copper nanoparticles [8] in water, and 10% enhancements were reported for as little as 0.005% (v/v) gold nanoparticles in water [4]. By contrast, Zhong and coworkers [8] report 35% enhancement in the thermal conductivity of nanofluids containing 0.8% (v/v) carbon nanotubes (CNT). As the thermal conductivity of CNT is about an order of magnitude higher than that of copper or gold, we would expect nanofluids containing copper or gold particles to exhibit lower enhancements than nanofluids containing CNTs, or for nanofluids containing CNTs to exhibit much larger enhancements than nanofluids containing copper or gold. Clearly, there are inconsistencies in the literature data. This is also apparent in the results of Li and coworkers [7] for 0.5% (v/v) copper particles in ethylene glycol (EG). Their work reports an increase in the thermal conductivity enhancement from about 10 to about 45% when the temperature increases from 10 to 50°C, but shows no increase in the thermal conductivity of EG with temperature. Finally, we note that many of these experiments employed very low volume fractions of nanoparticles. As a result, it is often difficult to separate size effects in these studies. Therefore, we have measured the thermal conductivity of nanofluids containing several volume fractions of metallic nanoparticles and report these results in the present work.

**Table 2:** Evaluation of the modified geometric mean thermal conductivity model

						**Size indep**.		**Size dep**.	
						
Particle	Fluid	φ/% v/v	*T*/K	*L*/nm	**Data Ref**.	AAD	*N*	AAD	*n*
Ag	Water	1-4 × 10^-1^	298	15	[3]	0.40	0.38	0.40	0.55
Ag + citrate	Water	1 × 10^-3^	303-333	70	[4]	2.99	1.00	3.25	1.00
Cu	EG	1-3 × 10^-1^	298	10	[2]	5.24	0.60	5.40	0.82
Cu	Water	2.5-7.5	298	100	[5]	2.15	0.06	2.10	0.08
Cu	PFTE	2-25 × 10^-1^	298	26	[6]	3.47	0.14	3.45	0.19
Cu	EG	3-5 × 10^-1^	278-323	7.5	[7]	7.07	0.39	6.75	0.61
Cu	Water	5-30 × 10^-2^	298	42.5	[8]	1.61	0.81	1.56	0.92
Cu	Water	2-9 × 10^-3^	298	25	[9]	6.27	0.77	6.24	0.93
Au + thiolate	Toluene	5-11 × 10^-3^	299-333	3.5	[4]	0.77	0.81	2.60	1.00
Au + citrate	Water	1.3-2.6 × 10^-3^	303-333	15	[4]	5.19	1.00	5.25	1.00

PFTE, perfluorotriethylamine

## Experimental

**Figure 2:**
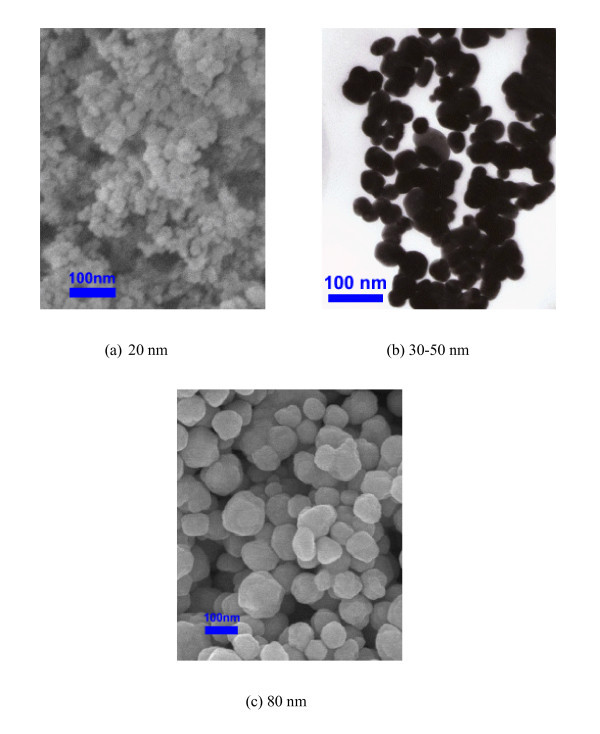
**SEM/TEM images of the silver nanoparticles provided by Nanostructured and Amorphous Materials, Inc. (Los Alamos, NM, USA)**. **(a) **20 nm, **(b) **30-50 nm, and **(c) **80 nm

Silver nanoparticles of sizes 20, 30 to 50, and 80 nm, loaded with 0.3 wt% polyvinylpyrrolidone (PVP), were purchased from Nanostructured and Amorphous Materials, Inc. (Los Alamos, NM, USA) and dispersed in EG to make nanofluids. The particle sizes were chosen to span sizes below and above the mean free path of electrons in silver. Scanning Electron Microscope (SEM) and Transmission Electron Microscope (TEM) images of the particles provided by the vendor are shown in Figure 2 and appear to show significant aggregation of the 20 nm particles. Nanofluids were prepared by dispersing pre-weighed quantities of nanoparticles into EG. The samples were subjected to ultrasonic processing to obtain dispersions. The nanofluid dispersions remained stable without any noticeable settling for over 2 h after processing.

The thermal conductivity of each nanofluid was measured using a liquid metal transient hot-wire device. The transient hot-wire method has been used successfully in our laboratory to measure the thermal conductivity of electrically conducting liquids [23] and nanofluids [10-14] over a broad range of temperatures. Our transient hot-wire device consists of a glass capillary, filled with mercury, and suspended vertically in the nanoparticle dispersion in a cylindrical glass cell. The glass capillary insulates the mercury 'wire' from the electrically conducting dispersion, and prevents current leakage when a voltage is applied to the 'wire'. The 'wire' is heated by application of a voltage and its resistance is measured using a Wheatstone bridge circuit with the 'wire' forming one arm of the circuit. The temperature change of the wire is computed from the resistance change of the mercury 'wire' with time. The data are used to calculate the effective thermal conductivity of the nanofluid via an analytical solution of Fourier's equation for a linear heat source of infinite length in an infinite medium. This solution predicts a linear relationship between the temperature change of the wire and the natural log of time, and this is used to confirm that the primary mode of heat transfer during the measurement is conduction. Corrections to the temperature are included for the insulating layer around the wire, the finite dimensions of the wire, the finite volume of the fluid, and heat loss due to radiation. The thermal conductivity is obtained from the slope of the corrected temperature-time line using the length of the mercury 'wire' in the calculation. An effective length of the wire that corrects for non-uniform capillary thickness and end effects is obtained by calibration with two reference fluids. In the present study, water and dimethyl phthalate were used as the reference fluids [24] and their properties were obtained from the literature [25]. Additional details of the apparatus and method are available elsewhere [23]. The experiment was performed five times for each sample and condition, and a data point reported in this work thus represents an average of five measurements with an estimated error of ±2%.

## Results

**Figure 3:**
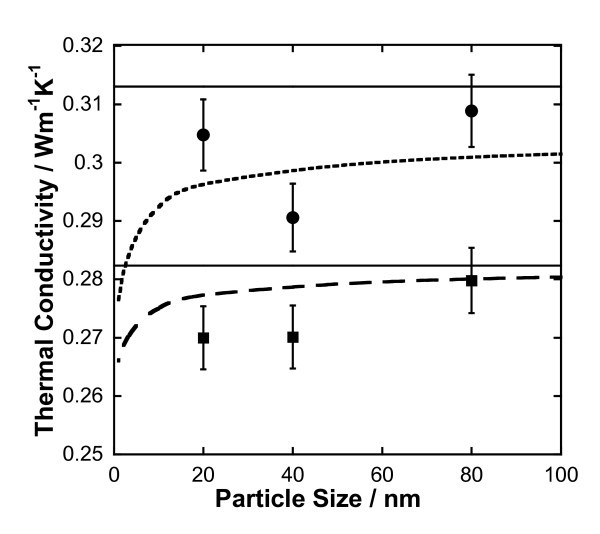
**Effect of particle size on the thermal conductivity of nanofluids containing silver nanoparticles**. *Points *(1% black square, 2% black circle) represent experimental data of this work. *Dashed *(1% ― ―, 2% ----) and *solid lines *represent calculated values assuming size dependence and without size dependence, respectively

Table 3 gives our measured values of the thermal conductivity enhancement for silver nanofluids. As noted previously, each data point represents the average of five measurements at a specific concentration and room temperature. The experimental data along with calculations using Equation 6 with and without considering the size dependence are presented in Figure 3. First, the size dependent model (Equations 3, 5, and 6 was used to correlate the data and a value of *n *= 0.088 was found to give the best fit with an AAD = 2.01%. Then, the same value of *n *was used in the size independent model (Equation 6) and resulted in an AAD = 3.64%. Figure 3 appears to confirm that the thermal conductivity of the nanofluid decreases with decreasing particle size, although the results are not conclusive. This could be due to the higher than expected thermal conductivity of nanofluids containing 20 nm silver particles resulting from aggregation (Figure 2a). Since the dry 20 nm particles were highly aggregated when purchased, we consider it likely that they are aggregated in the dispersion despite being subjected to sonication. In an aggregated structure, a fraction of the particles form a conductive pathway, which could result in enhanced conduction [26]. This is supported by numerical simulations and molecular dynamics studies [27-29]. On the other hand, the value of *n *= 0.088 obtained by fitting our data implies that the extent of aggregation was probably small and most particles were randomly dispersed in the fluid. Values of *n *close to ±1 in Table 2, obtained by fitting literature data, do not appear to be physically reasonable because they imply series or parallel alignment of particles.

**Table 3:** Thermal conductivity of nanofluids consisting of silver nanoparticles dispersed in ethylene glycol

*T*/K	φ/% v/v	*d*/nm	***k***_**EG**_**/W m**^**-1 **^**K**^**-1 **^[25]	***k***_**P**_**/W m**^**-1 **^**K**^**-1**^	***k***_**eff**_**/W m**^**-1 **^**K**^**-1**^	**Standard deviation in *k***_**eff**_
299.3	1	20	0.2544	123.49	0.2700	0.0052
299.9	1	30-50	0.2544	191.32	0.2701	0.0025
298.4	1	80	0.2544	263.50	0.2798	0.0023
300.8	2	20	0.2544	123.49	0.3048	0.0029
300.9	2	30-50	0.2544	191.32	0.2907	0.0023
300.5	2	80	0.2544	263.50	0.3089	0.0033

## Conclusions

A phenomenological model is presented for the thermal conductivity of metallic nanofluids that takes account of the size dependence of the thermal conductivity of metallic particles. The model was able to fit literature data for nanofluids using one adjustable parameter, although values of the fitted parameter were higher than expected. The thermal conductivity of nanofluids containing three sizes of silver nanoparticles dispersed in EG was measured and the data were fitted using our model. The results are in agreement with our previous work on nanofluids containing semiconductor or insulator particles, and appear to confirm that the thermal conductivity of silver nanofluids decreases with decreasing particle size.

## Abbreviations

CNT: carbon nanotubes; EG: ethylene glycol; PVP: polyvinylpyrrolidone.

## Competing interests

The authors declare that they have no competing interests.

## Authors' contributions

PW compiled the literature data, carried out experiments, proposed the thermal conductivity model, and participated in the writing of the manuscript. AST provided theoretical and experimental guidance, and participated in the writing of the manuscript. Both authors read and approved the final manuscript.
